# Maternal inheritance of F1 hybrid morphology and colony shape in the coral genus *Acropora*

**DOI:** 10.7717/peerj.6429

**Published:** 2019-02-19

**Authors:** Hironobu Fukami, Kenji Iwao, Naoki H. Kumagai, Masaya Morita, Naoko Isomura

**Affiliations:** 1 Department of Marine Biology and Environmental Science, Faculty of Agriculture, University of Miyazaki, Miyazaki, Miyazaki, Japan; 2 Akajima Marine Science Laboratory, Zamami, Okinawa, Japan; 3 Center for Environmental Biology and Ecosystem Studies, National Institute for Environmental Studies, Tsukuba, Ibaraki, Japan; 4 Sesoko Station, Tropical Biosphere Research Center, University of the Ryukyus, Motobu, Okinawa, Japan; 5 Department of Bioresources Engineering, National Institute of Technology, Okinawa College, Nago, Okinawa, Japan

**Keywords:** Scleractinia, Coral, Evolution, Hybrid, Intermediate morphology

## Abstract

**Background:**

The coral genus *Acropora* contains more than 150 species with very high morphological diversity. This high diversity may have been caused by repeated hybridization via mass spawning. However, we have little information whether hybrids are formed in these corals. Identifying morphological differences between hybrids and their parental species would provide an opportunity to find wild hybrids in the field and to understand how colony shapes of *Acropora* have become highly diversified throughout evolutionary history. In the two morphologically distinctive coral species *Acropora florida* and *A. intermedia* in the Indo-Pacific, their gametes show high rates of bi-directional intercrossing *in vitro*, and thus these two species are ideal species to investigate the morphological traits of the hybrids.

**Methods:**

We examined morphological characters of F1 hybrids from *A. florida* to *A. intermedia*, which were produced from *in vitro* crossing experiments. To compare morphological differences, we grew juveniles and mature colonies of reciprocal F1 hybrids (FLOint: *A. florida* eggs × *A. intermedia* sperm, and INTflo: *A. intermedia* eggs × *A. florida* sperm) and of the parental species (purebreds of *A. intermedia* and *A. florida*). We analyzed skeletal morphology such as colony size, branch length, and branching number, and compared them with those of a putative F1 hybrid between *A. florida* and *A. intermedia* found in the field. We also confirmed the molecular phylogenetic position of F1 hybrids, parental species, and a putative F1 hybrid using the mitochondrial non-coding region.

**Results:**

Our morphological analysis revealed that branching number of the F1 hybrids was intermediate relative to the parental species. Moreover, the FLOint hybrids were morphologically more closely related to the maternal species *A. florida*, and the INTflo hybrids were to *A. intermedia*. Molecular data showed that *A. florida* and *A. intermedia* were clearly divided into two clades, and that F1 hybrids grouped in the clade based on their maternal parent. A very similar pattern to the INTflo hybrids was obtained for the putative F1 hybrid in nature.

**Discussion:**

Our results revealed that F1 hybrids between two Indo-Pacific species *A. florida* and *A. intermedia* had intermediate morphology relative to their parent species but reflected the maternal parent more. Similarity to maternal species in hybrids is opposite to the Caribbean *Acropora* species that had more paternal morphological characters in hybrids. These results further suggest that some genetic factor in eggs is likely to affect determination of colony shape in the Indo-Pacific. At present, we have considered colonies with intermediate morphs between different species to be intra-specific morphological variation, but they may be real F1 hybrids. Indeed, a putative F1 hybrid represented similar morphological and molecular features to the F1 hybrids, and thus it is plausible to be attributed as a “real” F1 hybrid in nature.

## Introduction

Hybridization among species in the coral genus *Acropora* is presumed to occur due to the multi-specific synchronous spawning (i.e., mass spawning) events. Mass spawning in *Acropora* has been documented in the Indo-Pacific (Babcock et al., 1986). In Okinawa, Japan, it occurs in the early summer (Hayashibara et al., 1993), where *Acropora* species release egg-sperm bundles into the water. When these bundles reach the surface, they split apart into eggs and sperm, and fertilize with gametes released from other conspecific colonies. At the same time, gametes from other congeneric species are also present in the water column, which increases the risk of hybridization. So far, it has been reported that some *Acropora* species can hybridize *in vitro* crossing experiments: *Acropora milleopora* × *A. pulchra* (Willis et al., 1997, 2006), *A. formosa* (= *A. muricata*) × *A. nasuta* (Hatta et al., 1999), *A. tenuis* × *A. donei* × *A. yongei* (Fukami et al., 2003), *A. nobilis* (= *A. intermedia*) × *A. florida* (Hatta et al., 1999; Isomura, Iwao & Fukami, 2013; Isomura et al., 2016), *A. tenuis* × *A. loripes* (Chan et al., 2018), and *A. florida* × *A. sarmentosa* (Chan et al., 2018). Veron (1995) proposed that speciation and fusion (hybridization) have occurred repeatedly across evolutionary time scales in the scleractinian corals. Although these studies of hybridization in *Acropora* as above also support this hypothesis, no direct evidence for hybrids has been found in the Indo-Pacific. On the other hand, in the Caribbean, one species, *A. prolifera* is known to be a F1 hybrid between *A. cervicornis* and *A. palmata* (Vollmer & Palumbi, 2002).

Thus, in comparison with Caribbean *Acropora*, which are composed of only three species, even if hybrids exist in the Indo-Pacific, the high degree of range overlap, difficulty of species identification, and morphological diversity have made detecting natural hybrids a considerable challenge. Willis et al. (2006) reported on colony shapes of artificial F1 hybrids between *A. milleopora* and *A. pulchra*. The hybrids had morphologically intermediate shapes to the parental species, although there were no details describing the morphological characters. Juvenile hybrids outplanted to the field at 3 months of age showed heterosis with respect to growth and survival (Willis et al., 2006). They concluded that hybridization might play an important role in adaptation to new environments. Recently, Chan et al. (2018) also investigated the performance of F1 hybrids produced between *A. tenuis* × *A. loripes* and *A. florida* × *A. sarmentosa* in high temperature and pCO_2_ environments. They showed that some F1 hybrids tended to have higher survival and larger recruitment sizes than purebreds. On the other hand, morphological studies of F1 hybrids are limited due to technical difficulties in growing corals that take at least 3–5 years to reach adulthood in *Acropora* (Wallace, 1985; Iwao et al., 2010). At present, more than 150 congeneric species have been reported in *Acropora* (Wallace, 1999), but the species identification is exceptionally difficult due to the high morphological variation within and between species. Moreover, many congeneric species have overlapping ranges in general. For example, in Okinawa, at least 20 species inhabit a 500 × 500 m area as far as we investigated.

Previous studies have suggested that nested phylogenetic relationships among closely related species can be a result of reticulate evolution (fusion by hybridization) (Hatta et al., 1999; Van Oppen et al., 2001). However, incomplete lineage sorting or the retention of ancestral polymorphisms also could lead to a similar phylogenetic signal rather than present hybridization (Willis et al., 2006). Willis et al. (2006) also suggested that hybridization would occur rarely in the field, because inter-specific fertilization between the hybridizable species *A. millepora* and *A. pulchra* did not occur under the existence of both conspecific and heterospecific sperm. Thus, at present, it has been hypothesized that hybridization events are rare, even on evolutionary timescales. On the other hand, it is well known that many intermediate colony shapes exist between different species in *Acropora* in the field (Hatta & Matsushima, 2008). In fact, we have found a colony with intermediate colony shape between *A. florida* and *A. intermedia* in the field (Isomura, Iwao & Fukami, 2013), but these have been treated as intraspecific morphological variation in most cases. However, it remains a possibility that these are real hybrids, as Richards et al. (2008) suggested that rare species may be of hybrid origin. Recently, Kitanobo et al. (2016) reported that *A. florida* and *A. intermedia* breed preferentially with conspecifics at optimal gamete concentrations (10^6^ cells ml^−1^), but when sperm concentration was low (10^4^ cells ml^−1^), *A. florida* eggs displayed an increased incidence of fertilization by sperm of *A. intermedia*. This suggests that hybridization might occur in the field under the situation that conspecific colony density is low in the field, as the decline of coral coverage by bleaching events and outbreaks of the crown-of-thorns seastar *Acanthaster* spp. (Hoegh-Guldberg, 1999; Loya et al., 2001; Bruno & Selig, 2007; De’ath et al., 2012) may be reducing gamete concentrations in the water.

To investigate the presence of natural *Acropora* hybrids in the Indo-Pacific, it is essential to know the colony shapes and morphology of hybrids. One of the best ways is to grow hybrid offspring produced in experimental crosses, especially using two distinctive morphological species to compare the morphological characters between hybrids and parents. The following two species, *A. florida* and *A. intermedia*, have clearly differentiated colony shapes; *A. florida* has an irregular bottle brushing shape, while *A. intermedia* has a long branching colony shape. Our previous study showed that these two species hybridized *in vitro* with high fertilization rates; *A. florida* starts spawning 10–40 mins earlier than *A. intermedia*, and inter-specific fertilization rates between *A. florida* sperm and *A. intermedia* eggs were much higher than those of the reciprocal cross (Isomura, Iwao & Fukami, 2013). This is similar to hybridizing patterns in the Caribbean species, *A. cervicornis* (earlier spawner) and *A. palmata* (later spawner) (Fogarty, Vollmer & Levitan, 2012, also see Isomura, Iwao & Fukami, 2013). Hence, these two species, *A. florida* and *A. intermedia*, are an ideal combination to examine the morphology of hybrids.

In this study, we examined the morphology of F1 hybrid corals by analyzing morphological characters of parental and hybrid offspring produced in experimental crosses between *A. florida* and *A. intermedia*. These morphological analyses yield useful information on the process of colony formation, and are important for understanding the high diversity of colony shape in *Acropora*. In addition, the specimens used in the morphological analysis were analyzed phylogenetically using mitochondrial putative control region to confirm the phylogenetic position of F1 hybrids, a putative F1 hybrid, and parental species.

## Materials and Methods

### Preparing F1 hybrids

We performed *in vitro* crossing experiments in 2007 between *A. florida* and *A. intermedia* in the Akajima Marine Science Laboratory, Akajima Island, Okinawa, Japan. Collections of corals were permitted by Okinawa Prefectural Government (Permission number: 30-7). We then produced purebred larvae from the two parent species, and also two kinds of F1 hybrid larvae, which were produced from *A. florida* eggs × *A. intermedia* sperm (hereinafter called FLOint), and from *A. intermedia* eggs × *A. florida* sperm (INTflo). These hybrid and purebred larvae (more than 100 each) were kept separated in the water tank until they settled on the plates (see Isomura et al., 2016 in details). After settling, these plates were randomly transferred into cages, which were moored two m below the sea-surface and hanging by floats. The cages were located in a bay in Akajima Island that was six to seven m deep with a sandy bottom with no other corals nearby. We checked their growth 2 and 4 years later, and most of the living colonies were used for the following morphological analysis, but only three F1 hybrid colonies are currently alive (two of them spawned in 2014 and 2015; see Isomura et al., 2016). In addition, 10 purebreds (4-year-old) of each species were transplanted into the field in 2011. Only one colony survived in the field in 2014, but then they all died in 2016 due to a destructive typhoon.

### Morphological analysis

**Figure 1:**
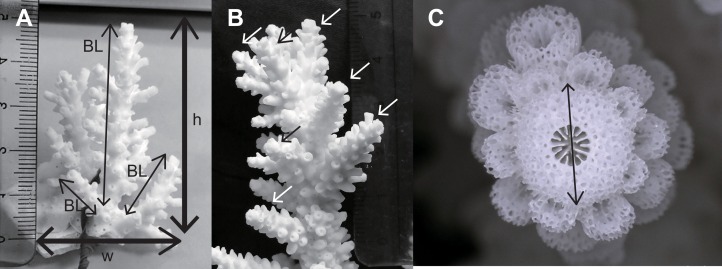
Skeletal characters for morphological analysis. (A) Height of a colony (h), width of a colony (w), branch length (BL). (B) Number of secondary branches per main branch. (C) Diameter of axial corallite.

We removed 10 4-year-old colonies from each of the four combinations (purebreds of two parent species, FLOint and INTflo F1 hybrids) from the cages, bleached them, and analyzed their skeletal morphology. For the morphological analyses, we used vernier calipers to measure maximum colony width, maximum colony height, the length of one to three main long branches in a colony (we used only branches that were more than 10 mm long), branching number (number of secondary branches on a main long branch), and the diameter of the axial corallite of each long branch (Fig. 1). We combined colony width and height as a measure of colony shape (height per width of colony). Values were averaged among branches for each colony for the other three characters.

We also observed colony shape, axial and radial corallites, and coenosteum structure of mature colonies (8 or 9 years old) for F1 hybrids. For parental species, we used 7-year-old colonies to describe their morphological features (see Isomura, Iwao & Fukami, 2013). Branching number was counted from the skeletons (one or two main branches were picked up and bleached for analysis to keep their specimens alive) of mature F1 hybrids (one colony each from FLOint and INTflo), and of mature purebreds of the two parent species (one colony each per species, because most purebreds died). In addition, we counted branching number and measured the branch length and diameter of axial corallites of branches >10 cm long (a part of a whole branch) that we broke up partially from colonies of *A. florida* and *A. intermedia* in the field. Moreover, branching number from only two branches (one collected in 2012 from the field, and another in 2015 that remained in the cage with F1 hybrids for 3 years) from a putative F1 hybrid that we found in the field were counted (same colony as *Acropora* sp. “int-flo” in Isomura, Iwao & Fukami, 2013).

### Statistical analysis

To build a classification model among purebreds and F1 hybrids (*A. florida*, *A. intermedia*, FLOint and INTflo F1 hybrids) based on morphological characters, we used the multinomial logit model with a logit link function, implemented with the “multinom” function of the “nnet” R package (Venables & Ripley, 2002). First, we selected the optimum classification model that included the fewest morphological characters to classify the purebreds and F1 hybrids among models. The model included colony shape, branch length, branching number, and diameter of axial corallites using 4-year-old colonies. The optimum classification model has the smallest Akaike information criterion (AICc) (the adjusted version of AIC for small sample sizes; Burnham & Anderson, 2002). This statistical procedure optimizes prediction performance, while classical statistical tests based on *p*-value find significance of effects (Burnham & Anderson, 2002). Second, we evaluated the optimum classification model examining colonies of all the different ages and wild colonies together, followed by repeating the analysis with 1,000 cross-validation steps using a randomly selected 2/3 of data to estimate model parameters. The remaining 1/3 of the data were used to evaluate classification performance of the model. As evaluation metrics for correct classification, we used overall accuracy, sensitivity (accuracy of positive classifications), specificity (accuracy of negative classifications), and True Skill Statistics (TSS: Allouche, Tsoar & Kadmon, 2006). Statistical analyses were performed using R version 3.5.0 (R Core Team, 2018).

### Mitochondrial analysis

We used the partial mitochondrial putative control region (mtCR; 759 bp of the total 1,200 bp; Van Oppen et al., 2002) to confirm the phylogenetic position of F1 hybrids, a putative F1 hybrid, and parental species. All samples were collected from Akajima Island and Sesoko Island in Okinawa, Japan. In total, one FLOint F1 hybrid, one INTflo F1 hybrid, one putative F1 hybrid, 16 colonies of *A. florida* (Akajima Island: 12; Sesoko Island: 4) and 15 colonies of *A. intermedia* (Akajima Island:14; Sesoko Island: 1) were analyzed. A small piece (5 × 5 mm) of coral tissue from each colony was digested in 1.5 ml CHAOS solution (modified guanidine solution: see Fukami et al., 2004) for several days. Total DNA was extracted from the CHAOS solution with phenol/chloroform extraction. PCR amplifications of the mtCR were conducted using the following primers: RNS2 5′-CAG AGT AAG TCG TAA CAT AG-3′, and GR 5′-AAT TCC GGT GTG TGT TCT CT-3′ (Suzuki et al., 2008). The protocol for amplification was 94 °C for 30 s, followed by 30 cycles at 94 °C for 20 s, 56 °C for 30 s, and 72 °C for 90 s, with a final extension of 72 °C for 5 min. PCR products were treated with shrimp alkaline phosphatase to inactivate dNTPs, and remnant primers were digested with exonuclease I at 37 °C for 40 min followed by 80 °C for 20 min. The DNA sequences were then determined via direct sequencing (FASMAC, CO, KANAGAWA, JAPAN). All DNA sequences obtained in this study were submitted to DDBJ (accession numbers LC428098–LC428131).

DNA sequences were aligned with Sequencher version 5.1 (Gene Codes, Ann Arbor, MI, USA) and SeaView version 4.3.0 (Gouy, Guindon & Gascuel, 2010). Phylogenetic trees were reconstructed using the neighbor-joining (NJ) and maximum-likelihood (ML) methods. For the NJ and ML methods, we assumed a model of nucleotide evolution obtained using the AIC, as implemented in MrModeltest 2.2 (Nylander, 2004). The most appropriate models of nucleotide evolution were generalised time reversible (GTR) with equal base frequencies. PAUP* (Swofford, 2002) was used to estimate the NJ topology and conduct a bootstrap analysis (bootstrap 1,000 times). PAUP was also used to reconstruct the best ML tree using a heuristic search and the tree-bisection-reconnection branch-swapping method. For outgroups, we used the genetically distinct *A. tenuis*, *A. latistella* and *A. papillare* (Van Oppen et al., 2001). We also included mtCR data of *A. florida* and *A. intermedia* from Australia (Van Oppen et al., 2001; Rosser et al., 2017).

## Results

### Morphology of young colony

**Figure 2:**
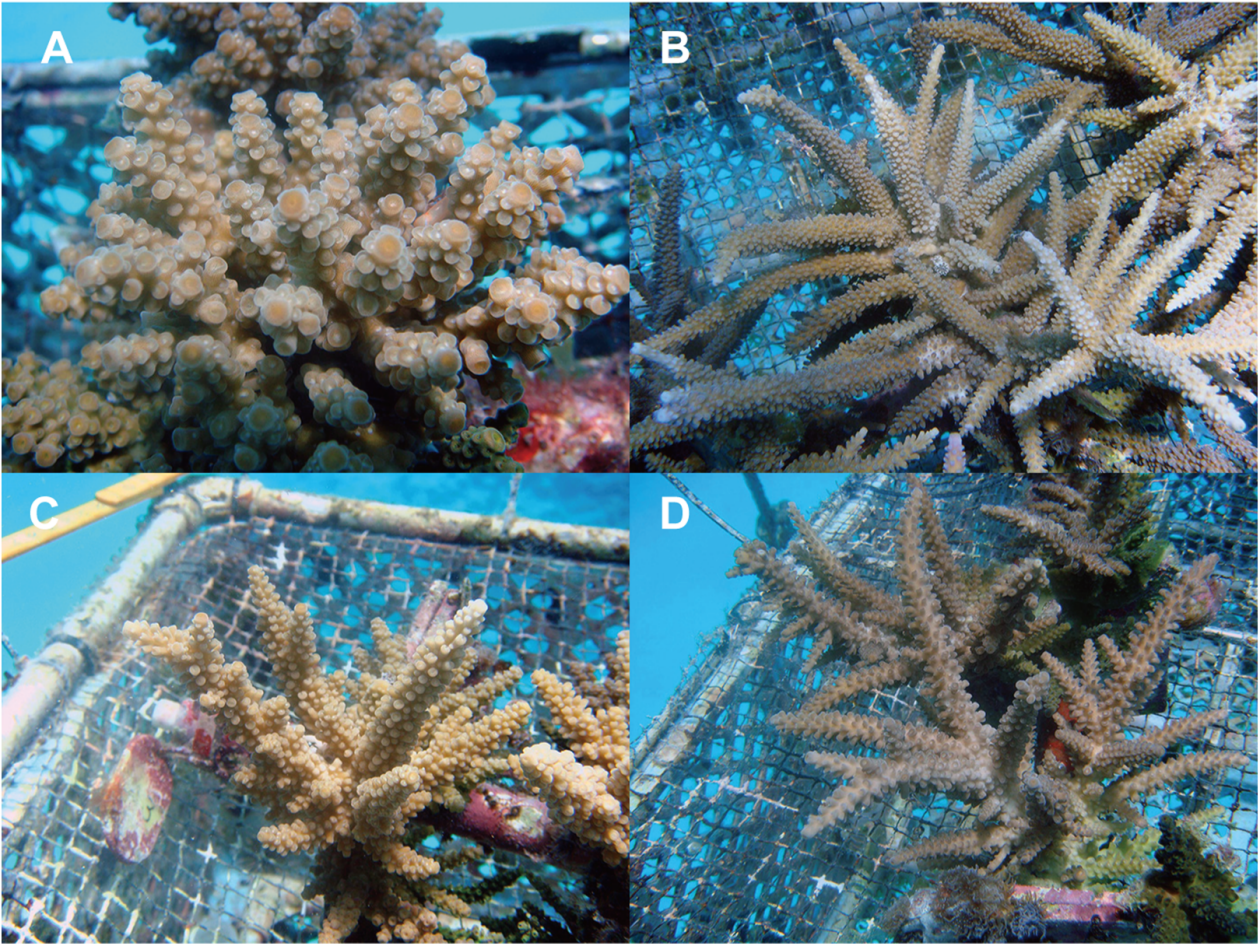
Living specimens of 2-year-old offspring. (A) Purebred juveniles of *A. florida* (flo). (B) Purebred juveniles of *A. intermedia* (int), (C) F1 hybrid juveniles of *A. florida* eggs × *A intermedia* sperm (FLOint). (D) F1 hybrid juveniles of *A. intermedia* eggs × *A. florida* sperm (INTflo).

Living specimens of 2-year-old offspring were observed based on the photos we took (Fig. 2). Purebreds of *A. florida* formed hispidose and cespitose forms, whereas purebreds of *A. intermedia* were an arborescent form. Hybrid offspring of FLOint were cespitose but with much longer branches than those of *A. florida*. Hybrid offspring of INTflo appeared similar to purebreds of *A. intermedia*, but they had more secondary branches.

**Figure 3:**
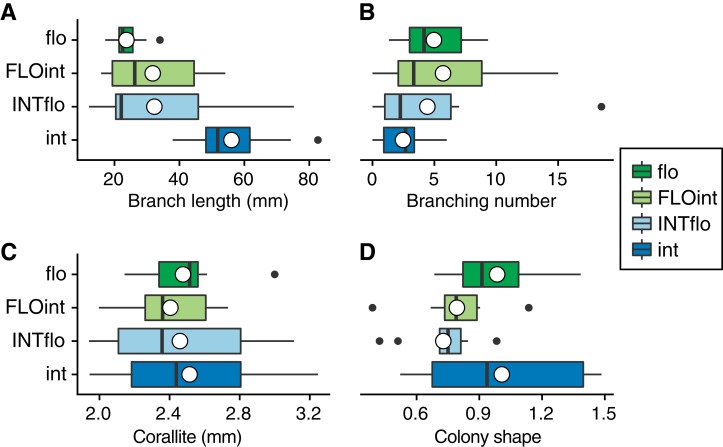
Morphological characters of 4-year-old offspring. (A) Branch length, (B) Branching number, (C) Diameter of axial corallite, (D) Shape of colony (height per width of colony). flo: *A. florida*. int: *A. intermedia*. FLOint: F1 hybrid of *A. florida* eggs × *A intermedia* sperm. INTflo: F1 hybrid of *A. intermedia* eggs × *A. florida* sperm. Box-and-whisker plots show the median (vertical bold line inside the box), mean (circle), interquartile range (box), range (whiskers) and outliers (dots).

**Figure 4:**
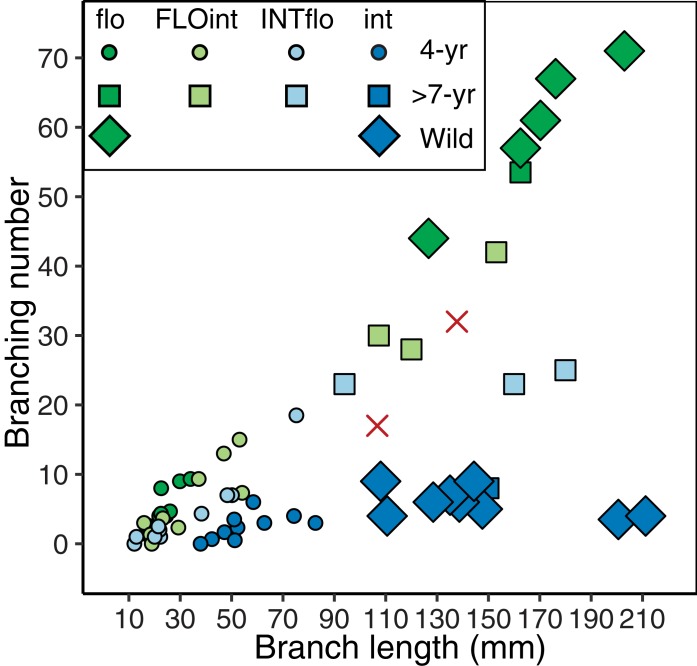
Relationship between branch length and branching number of 4-year-old offspring and matured colonies (7 and later years old). *A. florida* purebreds are shown in green, FLOint F1 hybrids in light green, INTflo F1 hybrids in light blue, and *A. intermedia* purebreds in blue. 4-year-old colonies are indicated with circles, and mature colonies are squares. Data from partial branches of wild colonies of *A. florida* and *A. intermedia* are shown in diamonds. Two branches from a putative wild F1 hybrid collected in the field are shown by the x-mark in red (shorter one collected in 2012, another in 2015).

In the skeletal morphological examination of 10 colonies for each 4-year-old offspring, we found large variation in their colony size and branch length (Table S1; Fig. 3). The width and height of purebreds of *A. intermedia* ranged from 36.0 to 140.5 mm, and from 38.8 to 79.8 mm, respectively. The colony shape (height per width of colony) ranged from 0.52 (73.3 mm high/140.5 mm wide in a colony) to 1.48 (79.8 mm high/53.8 mm wide in a colony). The branch lengths of *A. intermedia* purebreds were the longest and *A. florida* purebreds were the shortest, while the branch lengths of F1 hybrids were intermediate between those of purebreds of both parental species. On the other hand, axial corallite showed less variation, and were of similar sizes among the genotypes. The differences in the mean branching number were also small among the genotypes, but *A. intermedia* purebreds had the fewest branchings (mean 2.5 branchings) and the others had more branching (mean 5.0 branchings) (Table S1). For *A. intermedia* purebreds, branching number was saturated around six, even though the branch length became longer. However, for the others, it increased with the growth of the branch (Fig. 4).

### Morphology of matured colony

**Figure 5:**
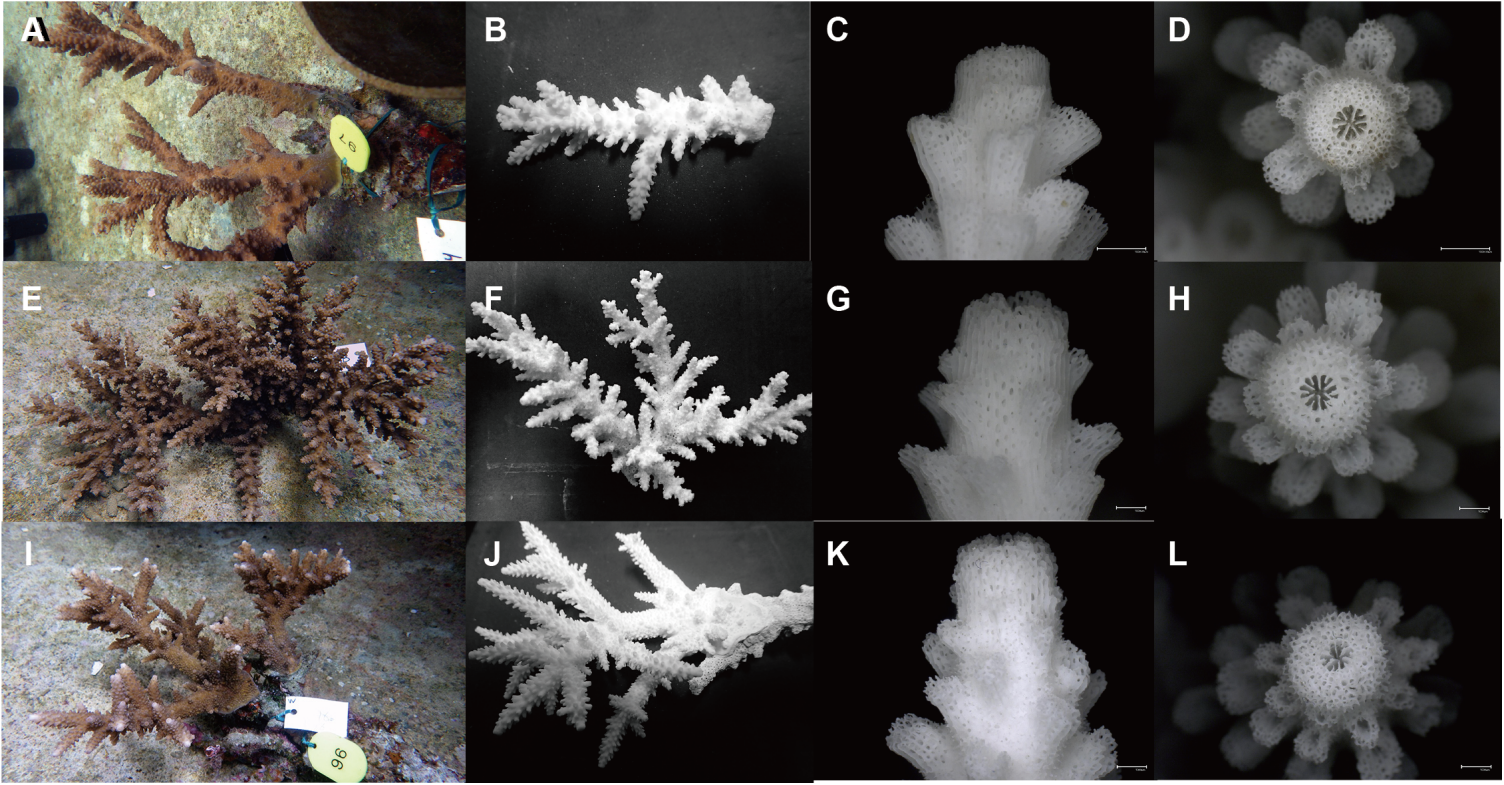
F1 hybrids of 8 or 9 years of age. (A–D) INTflo, (E–H), FLOint, (I–L), putative wild F1 hybrid from field (kept in same cage as F1 hybrids in 3 years). Bar: 1.0 mm.

Colony shape of FLOint and INTflo of F1 hybrids at 8 to 9 years old still appeared different in two parameters measured for colony shape and branching number (Fig. 5). Purebreds of parent species at 7-year-old produced a similar colony shape to the parent species (see Figs. 1c, d in Isomura et al., 2016).

Branching number and branch length of mature colonies of F1 hybrids and purebreds were compared with those of 4-year-old specimens (Fig. 4). Notably, the branching number of INTflo saturated around 25 in the mature colony. *A. florida* and FLOint increased proportionally to around 60 and 40, respectively, in mature colonies, because they formed a bottle brush shaped colony. For *A. intermedia*, branching number in mature colonies was twice as many as that at 4-year-old, even though the branch length in mature colonies was three times as long as that at 4 years old. For *A. florida* and *A. intermedia*, these patterns did not change even when additional data from partial branches collected in the field were added. For a putative F1 hybrid in the field, the relationship between branching number and branch length overlapped with the variation of FLOint and INTflo.

### Statistical analysis and classification model

**Figure 6:**
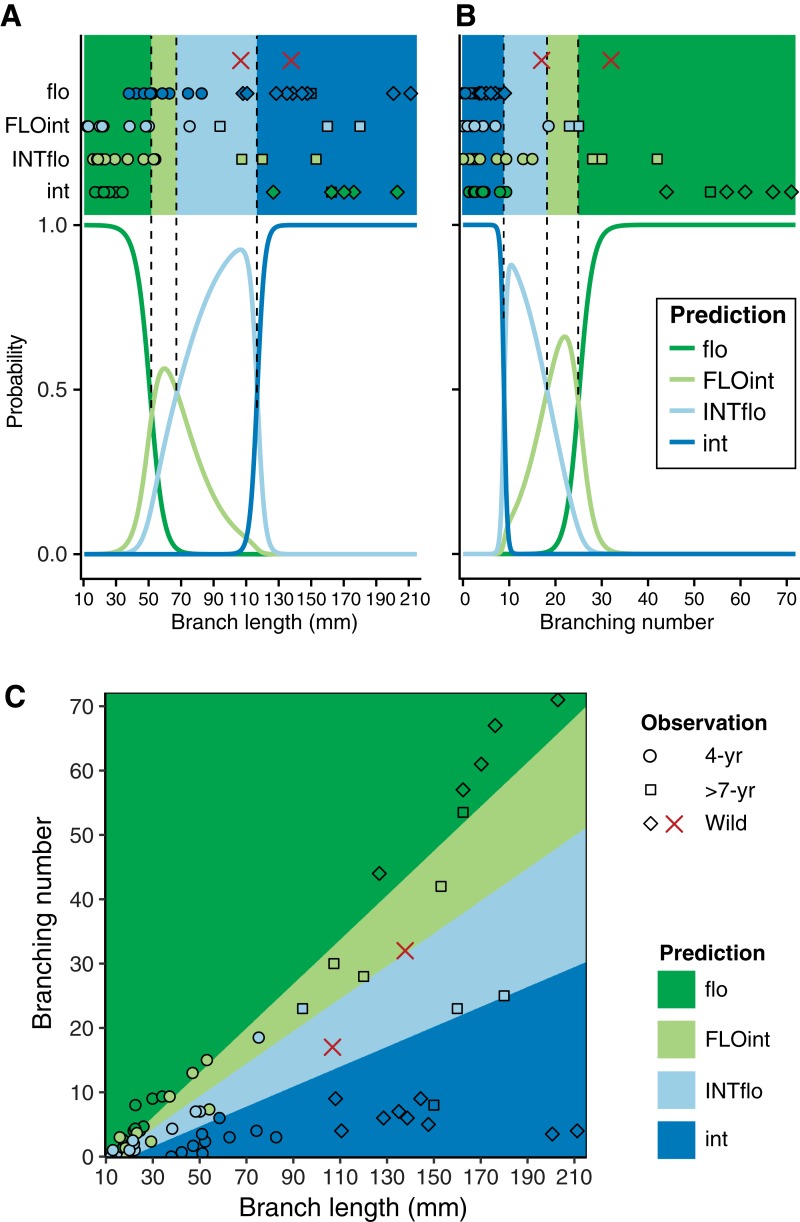
Observed and predicted classification among purebreds and F1 colonies. Colonies of all ages and putative wild F1 hybrids were used to build and evaluate the classification model (the multinomial logit model with 1,000 repeated cross-validation). (A) Predicted classification using the branch length, (B) predicted classification using branching number, (C) predicted classification using both branch length and branching number. Points indicate the observations plotted onto the predicted classification. In (A) and (B) predicted probability of each classification is also shown. flo: Purebred juveniles of *A. florida*. int: Purebred juveniles of *A. intermedia*. FLOint: F1 hybrid juveniles of *A. florida* eggs × *A intermedia* sperm. INTflo: F1 hybrid juveniles of *A. intermedia* eggs × *A. florida* sperm. Two branches from a putative wild F1 hybrid collected in the field are shown by x-mark in red (shorter one collected in 2012, another in 2015).

In statistical model comparisons, we found that the optimum combination of morphological characters that explained differences among purebreds and F1 hybrids of 4-year-old colonies was branch length and branching number, with significant differences in AICc value (ΔAICc = 3.2) against the second optimum model (Table S2). Regardless of their age, the correct classification of colony types based solely on the branch length or branching number was less than half (Fig. 6A: 33.3% by branch length; Fig. 6B: 33.1% by branching number), while the optimum classification model using both branch length and branching number (Fig. 6C; Table S3) correctly classified 67.4% of the genotypic differences (overall accuracy: 67.4 ± 7.6%; sensitivity: 61.9 ± 8.6%; specificity: 89.4 ± 2.4%; TSS: 0.51 ± 0.11). This model classified F1 hybrids as having intermediate morphological characteristics between their parental species (Fig. 6; Table S3): FLOint was more closely related to *A. florida* and INTflo was more closely related to *A. intermedia*. The optimum model classified one branch (collected in 2012) of a putative F1 hybrid as INTflo and the other branch (collected in 2015) as FLOint.

### Mitochondrial analysis

**Figure 7:**
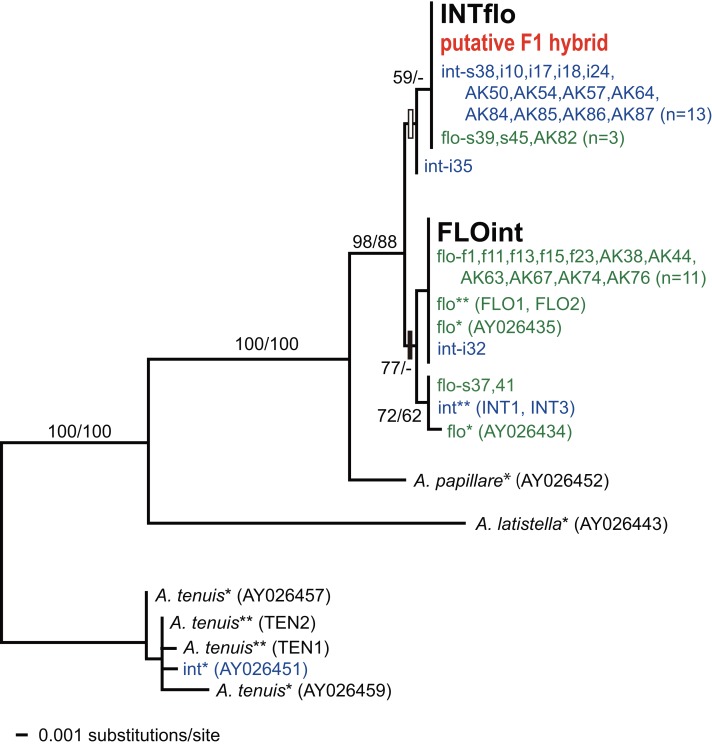
Molecular phylogenetic tree of *A. intermedia*, *A. florida*, F1 hybrids (INTflo and FLOint) and a putative F1 hybrid, based on the mitochondrial putative control region. Specimens from Australia are represented by one asterisk (Great Barrier Reefs: Van Oppen et al., 2001) or two asterisks (Western Australia: Rosser et al., 2017). White square indicates the existence of specific substitutions (Site# 711:T, 759:T) for the intermedia clade, and black square shows the existence of specific substitutions (711:A, 759:G) for florida clade. Bootstrap values (NJ/ML) are shown on the main branches. Colony number or accession number are shown in parentheses. int: *A. intermedia*, flo: *A. florida.* Letters (AK, f, or i for Akajima, s for Sesoko) and numbers following int or flo indicate our specimen’ sample numbers.

The phylogenetic tree showed that our *A. florida* and *A. intermedia* were genetically separated into to two distinct clades (florida and intermedia clades), with some exceptions; four out of the 20 (four from Australia) *A. florida* colonies grouped in the intermedia clade, and three of 18 (three from Australia) *A. intermedia* colonies were included in the florida clade. F1 hybrids were placed in the clade containing their maternal species (Fig. 7). A putative F1 hybrid was included in the intermedia clade with INTflo. In this tree, *A. intermedia* from the Great Barrier Reef (GBR) in Australia was genetically distinct from those in Japan and western Australia, but *A. florida* from Australia (GBR and western region) grouped in the clade with the other *A. florida* specimens in Japan.

## Discussion

### Morphological evolution

In animals and plants, hybrids often have intermediate morphological characteristics relative to their parental species (Richards & Hobbs, 2015). In corals, colonies with morphology that is intermediate between species also have been reported (Hatta & Matsushima, 2008; Isomura, Iwao & Fukami, 2013), suggesting they may be the result of hybridization. In this study, our results clearly showed that F1 hybrids have intermediate morphological characters in branching patterns (i.e., number of secondary branches) and branch length. Moreover, colony shape of F1 hybrids seems to be inherited maternally. Willis et al. (2006) also showed a juvenile colony of hybrids between *A. pulchla* eggs × *A. millepora* sperm, which more closely resemble the maternal parent (*A. pulchra*). Thus, colony morph seems to be largely determined genetically and/or by cytoplasmic inheritance and less so by environmental factors. In contrast, in the Caribbean *Acropora*, the colony morphs of F1 hybrid *A. prolifera* are inherited paternally; *A. prolifera* with bushy colony-morph had a mitochondrial background of *A. palmata* with a palmate colony-morph, whereas *A. prolifera* with a palmate colony-morph had a mitochondrial background of *A. cervicornis* with a bushy colony-morph (Vollmer & Palumbi, 2002). They also suggested maternal and/or cytoplasmic effects accounted for these morphological differences in *A. prolifera*, but this pattern was opposite that in the Indo-Pacific populations. At present, it is unknown why inheritance of colony morph from parents to F1 hybrids differs between Indo-Pacific and Caribbean regions. Genes affecting colony morphs might exist in corals, although there are only three cases (including this study) that report hybrid morphology. To understand the high morphological diversity of *Acropora* species, genome analysis with many loci will be needed. Future studies should perform crossing experiments to grow artificial F1 hybrids.

The relationship between branching number and branch length was a key character that separated *A. florida* and *A. intermedia*, so it may be better to investigate this relationship in the field to find F1 hybrids. In fact, a putative F1 hybrid that we found in the field (Isomura, Iwao & Fukami, 2013) showed intermediate patterns between *A. florida* and *A. intermedia.* Furthermore, whole colony shape of this candidate looks like INTflo rather than FLOint (Fig. 5), because they do not form a bottle brushing colony shape. However, it was not different enough from FLOint and INTflo in branching number and branch length (Figs. 4 and 6C). In addition, this candidate was grouped with the intermedia clade with INTflo. Although it is possible that the candidate is classified as an undescribed species with intermediate morphological characters between *A. intermedia* and *A. florida*, this candidate deserves to be described as a natural F1 hybrid between *A. intermedia* eggs × *A. florida* sperm in the field. So far, we have never found additional colonies of this candidate in the field on Akajima Island. Therefore, even though this candidate was an F1 hybrid, it must be very rare in the field.

Although most morphological characters, such as branching pattern are variable among species in *Acropora*, morphology of axial corallite might be a good character to define hybridizing species groups. Wallace (1999) used phylogenetic analysis of 24 species groups of *Acropora* based on morphological characters (i.e., branching, colony morphs, etc). In this “morphology based tree”, two species, *A. intermedia* and *A. florida* are in the different morphological groups “robusta” and “florida”, respectively. However, their axial corallites are quite similar with regard to size and shape as far as we observed. Therefore, axial corallite morphology might be a reliable-candidate to be grouped among other hybridizing species.

### Phylogenetic relationships of *A. florida* and *A. intermedia*

Mitochondrial phylogenetic trees showed that *A. intermedia* and *A. florida* were separated from each other with a few exceptions, and F1 hybrids were included in the maternal clade. This genetic relationship is similar to that of three Caribbean *Acropora* species, *A. cervicornis*, *A. palmata*, and *A. prolifera*, shown in Vollmer & Palumbi (2002). Isomura, Iwao & Fukami (2013) showed that the hybridizing pattern between *A. intermedia* and *A. florida* is very similar to that of *A. cervicornis* and *A. palmata*; fertilization rates are much higher in the combination of sperm of earlier spawner (*A. florida*, or *A. cervicornis*) and eggs of later spawner (*A. intermedia*, or *A. palmata*) than in the reciprocal combination. Considering that a chance to encounter hybridizable gamates is limited due to rapid sperm dilution in the field (Omori et al., 2001), as we suggested previously (Isomura, Iwao & Fukami, 2013; Isomura et al., 2016), *A. intermedia* and *A. florida* would hybridize rarely in the field just as the Caribbean species do.

As shown in Fig. 7, mitochondrial haplotypes of *A. intermedia* from the GBR in Australia was genetically distant from ones from Japan. Although misidentification of species might occur for this species, this species has several specific morphological characters, such as large axial corallite and two different types of radial corallites, which make this species easy to identify. *A. intermedia* from the GBR in Australia were placed phylogenetically in the same clade in an early hour spawning group that included *A. tenuis* and *A. donei* (see Fukami et al., 2003). In Japan, however, all data from *A. intermedia* were not included in the early hour spawning group. Rather, this species was closely related to *A. florida*. This phylogenetic position seen in Japanese samples were also confirmed in phylogenetic trees using nuclear (single nucleotide polymorphism (SNP) and *PaxC*) and mitochondrial (mtCR, Fig. 7) markers, using *A. intermedia* from the western coast of Australia (Rosser et al., 2017). Based on these data, the phylogenetic position of *A. intermedia* is likely closely related to *A. florida*, but not *A. tenuis*. On the other hand, molecular phylogenetic analyses by Rosser et al. (2017) revealed that several species of *Acropora* (e.g., *A. donei*, *A. digitifera*, and *A. lutkeni*) contained cryptic species. To determine whether they are cryptic species or not, and to reduce the possibility of misidentification of species, more samples from the GBR will need to be analyzed in the future.

## Conclusions

Our results reveal that F1 hybrids of *A. florida* and *A. intermedia* represent intermediate morphology between parent species, and that the morph is more similar to the maternal species, which differs from the Caribbean *Acropora* hybrid, which is similar to the paternal species. This finding provides reliable morphological characters to identify hybrids in nature and will provide valuable information to understand evolutionary processes driving the morphological diversity in *Acropora* in the Indo-Pacific. Moreover, a putative F1 hybrid, which we considered as a wild F1 hybrid between *A. florida* and *A. intermedia*, was morphologically and genetically similar to our artificial F1 hybrids (colonies from *in vitro* crossing). Therefore, this candidate would be a natural F1 hybrid of *A. florida* and *A. intermedia*. Despite the potential for coral hybrids in nature, details on their morphology (even those cultured in aquaria), are limited (Vollmer & Palumbi, 2002; Willis et al., 2006; this study). This may be because hybrids are difficult to culture in aquaria. For example, we have tried unsuccessfully several times to grow F1 hybrids in aquaria, which die within 2 years due to predation by snails, disease, and sudden death. Although culture of hybrids is technically difficult, more data are needed from different combinations of intercrossing species to find wild hybrids in the Indo-Pacific. The continuous study of hybrids may reveal how many species have been affected through hybridization.

## Supplemental Information

10.7717/peerj.6429/supp-1 Supplemental Information 1 Summary of measurements of morphological characters of four-year-old offspring used in the morphological analyses.

10.7717/peerj.6429/supp-2 Supplemental Information 2 Classification model among the purebreds and F1 hybrids of four-years old, using a multinominal logit model.

10.7717/peerj.6429/supp-3 Supplemental Information 3 Coefficients of classification model among the purebreds and F1 hybrids of all age and wild colonies, using multinominal logit model with 1000 repeated cross-validation.

10.7717/peerj.6429/supp-4 Supplemental Information 4 Raw data of morphological characters of *Acropora intermedia*, *A. florida*, F1 hybrids, and a putative F1 hybrid. (A) *A. intermedia*. (B) *A. florida*. (C) FLOint F1 hybrid. (D) INTflo F1 hybrid. (E) A putative F1 hybrid.

## Additional Information and Declarations

### Field Study Permissions

The following information was supplied relating to field study approvals (i.e., approving body and any reference numbers): Collections of corals were permitted by Okinawa Prefectural Government (Permission number: 30-7.)

### DNA Deposition

The following information was supplied regarding the deposition of DNA sequences: The partial mitochondrial putative control region sequences for phylogenetic analysis described here are accessible via DDBJ accession numbers LC428098–LC428131.

### Data Availability

The following information was supplied regarding data availability: The raw data is available in Tables S1–S3.
