# Supplementary material for: Maternal inheritance of F1 hybrid morphology and colony shape in the coral genus Acropora
Source: PeerJ. 2019 Feb 19;7:e6429. doi: 10.7717/peerj.6429 (PMC6385702; doi:10.7717/peerj.6429)
Supplement: Supplemental Information 1 [file peerj-07-6429-s001.docx]

**Supplementary Table 1.** Summary of measurements of morphological characters of four-year-old offspring used in the morphological analyses. Maximum values are shown in bold, and minimum values are shown by mesh. flo: *A. florida*. int: *A. intermedia*. FLOint: F1 hybrids of *A. florida* eggs × *A intermedia* sperm. INTflo: F1 hybrids of *A. intermedia* eggs × *A. florida* sperm.

int flo INTflo FLOint

Total No. 10 10 10 10

**Colony:**

Width (mm)

mean±SD **70.56±34.31** 35.50±11.68 47.62±22.94 49.44±20.45

Min–Max 36.02–140.48 15.85–52.83 27.25–91.03 26.45–95.56

Height (mm)

mean±SD **61.99±15.26** 32.93±7.17 34.69±17.71 41.34±23.32

Min–Max 38.83–79.80 21.91–44.68 10.60–79.93 13.56–67.86

Colony shape (height per width)

mean±SD **1.01±0.37** 0.99±0.23 0.73±0.18 0.79±0.18

Min–Max 0.52–1.48 0.68–1.38 0.42–0.98 0.39–1.14

**Branch:**

Length (mm)

mean±SD **56.02±13.93** 23.70±5.24 32.28±20.30 32.15±14.62

Branching No.

Mean 2.5±1.8 5.0±2.8 4.4±5.5 **5.7±5.2**

Min–Max 0–6 1.3–9.3 0–18.5 0–15

Axial corallites

Diameter (mm) **2.51±0.42** 2.48±0.25 2.46±0.43 2.40±0.25
