# Supplementary material for: Maternal inheritance of F1 hybrid morphology and colony shape in the coral genus Acropora
Source: PeerJ. 2019 Feb 19;7:e6429. doi: 10.7717/peerj.6429 (PMC6385702; doi:10.7717/peerj.6429)
Supplement: Supplemental Information 2 [file peerj-07-6429-s002.docx]

**Supplementary Table 2.** Classification model among the purebreds and F1 hybrids of four-years old, using a multinomial logit model. Ranking of classification models based on AICc is shown. Models with ΔAICc less than five and null model (intercept only) are displayed. BL: Branch length, BN: Branching number, CS: colony shape (length per width of colony).

| Model | logLik | AICc | ΔAICc |
| --- | --- | --- | --- |
| BL+BN | –26.0 | 76.1 | 0 |
| BL+BN+CS | –21.9 | 79.3 | 3.2 |
| Null | –55.5 | 117.6 | 41.5 |
