# Supplementary material for: Maternal inheritance of F1 hybrid morphology and colony shape in the coral genus Acropora
Source: PeerJ. 2019 Feb 19;7:e6429. doi: 10.7717/peerj.6429 (PMC6385702; doi:10.7717/peerj.6429)
Supplement: Supplemental Information 3 [file peerj-07-6429-s003.docx]

**Supplementary Table 3**. Coefficients of classification model among the purebreds and F1 hybrids of all age and wild colonies, using multinomial logit model with 1000 repeated cross-validation. The flo was used as the basal group. Estimates [2.5%, 97.5% quantiles] are shown. BL: Branch length, BN: Branching number, CS: colony shape (length per width of colony). flo: *Acropora florida*, int: *A. intermedia*, if: F1 hybrid of eggs of *A. intermedia* and sperm of *A. florida*, fi: F1 hybrid of eggs of *A. florida* and sperm of *A. intermedia*

|  | FLOint | INTflo | int |
| --- | --- | --- | --- |
| Intercept | –3.79  [–7.34, –2.17] | 0.312  [0.202, 0.576] | –0.897  [–1.62, –0.587] |
| BL | –5.02  [–8.94, –3.17] | 0.410  [0.287, 0.684] | –1.29  [–2.15, –0.898] |
| BN | –73.7  [–178, –23.0] | 3.92  [1.14, 9.40] | –23.9  [–59.6, –5.80] |
